# Systemic Th17 response in the presence of periodontal inflammation

**DOI:** 10.1590/1678-7757-2019-0490

**Published:** 2020-04-03

**Authors:** Lina J. Suárez, Daniel E. Vargas, Adriana Rodríguez, Roger M. Arce, Nelly S. Roa

**Affiliations:** 1 Universidad Nacional de Colombia Bogotá Colombia Universidad Nacional de Colombia, Bogotá, Colombia.; 2 Pontificia Universidad Javeriana Facultad de Odontología Centro de Investigaciones Odontológicas Bogotá Colombia Pontificia Universidad Javeriana, Facultad de Odontología, Centro de Investigaciones Odontológicas, Bogotá, Colombia.; 3 The Dental College of Georgia at Augusta University Department of Periodontics Augusta United States The Dental College of Georgia at Augusta University, Department of Periodontics, Augusta, United States.

**Keywords:** Th17 cells, Periodontal diseases, Autoimmunity, CD161 antigen, Interleukin-23

## Abstract

The relationship between periodontitis and the pathogenesis of other inflammatory diseases, such as diabetes, rheumatoid arthritis and obesity has been an important topic of study in recent decades. The Th17 pathway plays a significant role in how local inflammation can influence systemic inflammation in the absence of systemic pathology.

**Objective:**

To determine Th17 biased-cells in systemically healthy patients in the presence of generalized chronic periodontitis.

**Methodology:**

A total of 28 patients were recruited without systemic inflammatory pathology, which was determined by clinical history, the Health Assessment Questionnaire (HAQ) and rheumatoid factor detection. Of these patients, 13 were diagnosed as healthy/gingivitis (H/G) and 15 as generalized chronic periodontitis (GCP). Th17 (CD4^+^CD161^+^) cells and Th17IL23R^+^ (CD4^+^CD161^+^IL-23R^+^) cells were quantified by flow cytometry, based on the total cells and on the lymphocyte region, termed the “enriched population” (50,000 events for each).

**Results:**

The percentages of Th17 cells of the H/G and periodontitis groups were similar on total cells and enriched population (19 vs 21.8; p=4.134 and 19.6 vs 21.8; p=0.55). However, Th17IL23R+ cells differ significantly between periodontally healthy patients and generalized chronic periodontitis patients in both total cell (0.22% vs 0.65%; p=0.0004) and enriched populations (0.2% vs 0.75%; p=0.0266).

**Conclusions:**

GCP patients (otherwise systemically healthy) were characterized by increased Th17-proinflammatory cell phenotype positive for the IL-23 receptor in peripheral blood. The proportion of Th17 cells that are negative for the IL-23 receptor in the peripheral blood of systemically healthy patients seemed to be unaffected by the presence or absence of chronic periodontitis.

## Introduction

Periodontitis, currently recognized as a chronic inflammatory disease, has been linked to many other proinflammatory pathologies. During inflammation pathogenesis, naïve T helper (Th) cells are activated by recognition of a peptide antigen–class II major histocompatibility complex presented by antigen-presenting cells and their interaction with the T-cell receptor. After activation, Th cells divide and give rise to different clones of CD4^+^ effector cells, which can be divided into three main types; Th type-1 (Th1), Th type-2 (Th2) and Th type-17 (Th17) cells.^1^ Each type of effector cell phenotype presents different profiles of cytokine secretion eliciting unique functional characteristics during the inflammatory response.

Th17 cells differentiate into subprofiles with functions determined by the cytokines in the environment and characterized by the expression of surface markers. CD161, also known as killer cell lectin-like receptor B1 (KLRB1), is a marker associated with the pathogenesis of inflammatory diseases^2^ by T-cell proliferation and induction of Th1 cytokine secretion. CD161 is a marker of all human T-cells producing IL-17. Th17 cells originate from the subpopulation of CD4^+^CD161^+^ cells that express RORC2 and IL-23R.^3^^,^^4^ The naïve CD4+ initially differentiates into Th17 in the presence of TGF-b and IL-6. However, subsequent exposure to IL-23 is required for functional maturation, the maintenance of their phenotype and the acquisition of pathogenic functions. IL-23 regulates the overexpression of IL-23R directly or indirectly through TGF-b3 to increase its own signal and induce functionally mature Th17 pathogenic cells. In the absence of IL-23 signal, Th17 cells differentiate into non-pathogenic Th17-producing IL-10.^5^

Th17 cells play a central role in many inflammatory diseases, such as rheumatoid arthritis, ankylosing spondylitis, stomach cancer, chronic obstructive pulmonary disease,^6^^–^^12^ and periodontitis. Recently, it has been demonstrated in an animal model that the expansion of Th17 cells^13^ and the conversion of Foxp3+ T cells to exFoxp3Th17 cells^14^ are associated with the periodontitis pathogenesis. A significantly greater increase in IL-23 receptor expression was found in tissues with periodontitis compared with healthy tissues, thus confirming the importance of Th17/IL-23 axis activation in periodontal inflammation.^15^

The evidence of low magnitude inflammation in a systemic level caused by local infections in the periodontium is the key link between periodontitis and other systemic inflammatory diseases. Th17 subpopulations could be mediating this important effect.^16^^,^^17^ Th17 cell populations in the blood could potentially procure or perpetuate inflammation in patients with periodontal disease and could also eventually constitute a biological marker, which explains disease progression or activity.^16^^,^^18^ The study of Th17 populations may also explain how local infections in the periodontium influence the progression and prognosis of other related systemic diseases. Therefore, this study aimed to compare the percentages of Th17 (non-pathogenic) and Th17IL-23R^+^ (pathogenic) cells in systemically healthy patients in the presence of generalized chronic periodontitis.

## Methodology

### Patient selection

After institutional review board (IRB) approval (Universidad Nacional CIE-107-13), a sample of patients attending Universidad Nacional Dental School, who met the following inclusion criteria, was selected: 18+ years of age, systemically healthy, and having at least 20 teeth. The clinical periodontal examination was performed by probing six surfaces around the teeth to determine pocket depth, bleeding on probing and clinical attachment level. The periodontal diagnosis was confirmed *per* the criteria of the American Academy of Periodontology^19^ and further classified as healthy/plaque-induced gingivitis (H/G) or generalized chronic (mild, moderate or severe) periodontitis (GCP).^20^ Patients were excluded if they were pregnant or lactating, had systemic diseases (controlled or uncontrolled), received antibiotics, anti-inflammatory or corticosteroid therapy within 6 months prior to consultation (or required antibiotic prophylaxis prior to dental treatment) or were rheumatoid factor-positive. All recruited patients were surveyed to determine clinical manifestations of rheumatoid arthritis per the Health Assessment Questionnaire (HAQ).^21^^,^^22^ The rheumatoid factor was detected by the latex method (HumaTex RF, LabMark, Praha, Czech Republic). Absence of agglutination was considered negative (RF<12 IU/mL).

### Peripheral blood collection, antibody staining and flow cytometry analysis

**Figure 1:**
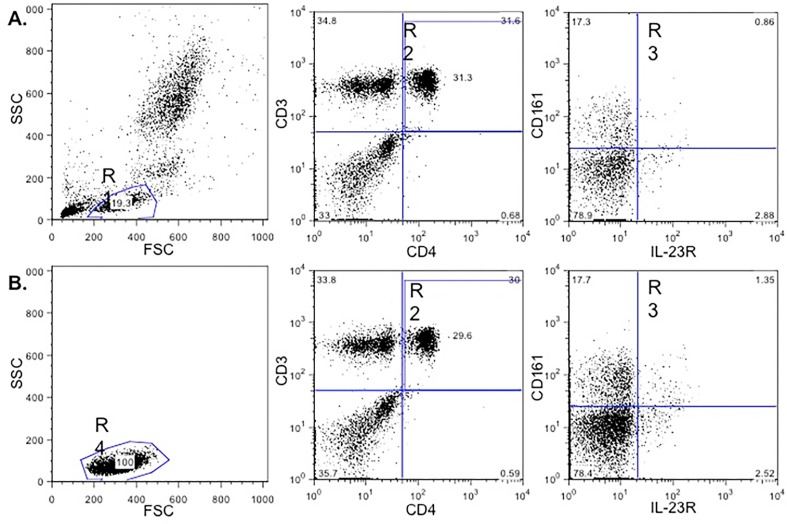
Analysis of cell populations by flow cytometry. A dot plot of a representative sample is shown. A: Analysis of total cells (acquisition of 50,000 events of all population), R1: Lymphocytes region. R2: CD3^+^CD4^+^ cells. R3: CD161^+^IL-23R^+^ cells; B: Analysis of enriched populations of lymphocytes. R4: Enriched lymphocytes region (acquisition of 50,000 events of this population), the information on the CD4^+^CD161^+^IL-23R^+^ cells was obtained in the same way as was done for the total cells

After obtaining patient informed consent, 50 mL peripheral blood was obtained by venipuncture (Beckton-Dickinson, Franklin Lakes, NJ, USA). Blood with ethylenediaminetetraacetic acid (EDTA) (Beckton-Dickinson, Franklin Lakes, NJ, USA) was used to quantify Th17 (CD3^+^CD4^+^CD161^+^) and Th17IL-23R^+^ (CD3^+^CD4^+^CD161^+^IL23R^+^) cells with anti-human monoclonal antibodies coupled with the following fluorochromes: anti-CD3 PerCP (T cells), anti-CD4 FITC (Th cells), anti-CD161 APC (marker of all human IL-17 producing T cells)^2^ (BD Biosciences, Pharmingen, San Diego, CA, USA) and anti-IL-23R PE (selective expression of IL-23 receptor for all human IL-17 producing T cells)^2^ (R&D Systems, Minneapolis, MN, USA). CANTO II Flow Cytometer (Beckton-Dickinson, San José, CA, USA) was used to analyze the cells. Data were analyzed using Flowjo 8.7 (FlowJo LLC, Ashland, OR, USA). Cell acquisitions were performed based on the total cells (50,000 events for all) and on the lymphocyte region, which we termed the “enriched population of lymphocytes” (50,000 events for each region) (Figure 1). For each acquisition, all events and enriched populations of lymphocytes were analyzed and the value *per* group was obtained in percentages (%) of Th17 and Th17IL-23R^+^ cells. The averages of these populations *per* patient group were then determined. The mean fluorescence intensity for the CD161 and IL-23R marker expressions were also analyzed.

### Statistical analysis

Non-parametric data distribution was observed (Shapiro-Wilk p<0.05). Data was reported as a median value [interquartile range]. Groups were statistically compared by Mann Whitney U test (p<0.05) using GraphPad Prism 8 software (San Diego, CA, USA).

## Results

A total of 28 patients (9 women and 19 men) ranging between 19 and 73 years of age with an average age of 34 were recruited. Patients had no relatable autoimmune disease. All patients answered the HAQ questionnaire to rule out symptoms associated with rheumatoid arthritis. Of these, 13 patients (6 women and 7 men) were included in the H/G group and 15 (3 women and 12 men) were included in the GCP group. The average age was significantly lower in the H/G group compared with the GCP group (Table 1).

**Table 1:** Age average and clinical scores of study groups

Parameters	H/G group	GCP group
Age	25.8±5.9	43.7±13.4
Probing depth	2.06±0.36 mm	3.13±0.86 mm
Clinical attachment level	0.59±0.32	3.66±1.37
Bleeding on probing	39.1%	77.7%

H/G group: healthy/plaque-induced gingivitis

GCP group: generalized chronic periodontitis

**Figure 2:**
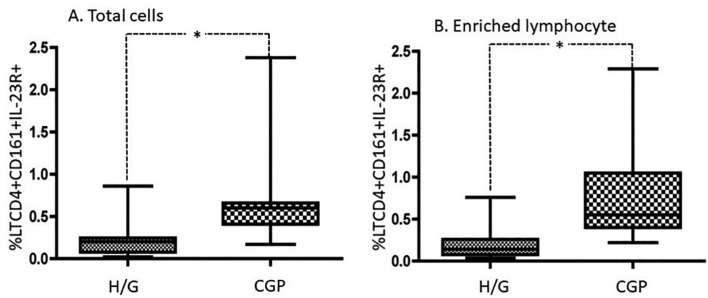
Comparative analysis of the percentage of CD4^+^CD161^+^IL-23R^+^ T lymphocytes between healthy/gingivitis patients (H/G) and diagnosed with generalized chronic periodontitis (GCP) on total cells (A) and enriched population of lymphocytes (B). Comparative analysis was performed using the Mann-Whitney U test. *p<0.05

Analysis of the Th17 cell population of the total cells showed no difference in the percentages between the H/G and GCP groups (19±5.1% *vs* 21.8±6.8%, p=0.4336). Similarly, no differences were found in the enriched lymphocyte population percentages (19.6±5.7% *vs* 21.8±6.705%, p=0.55). However, significant differences were found when comparing Th17IL-23R^+^ cells between the groups with respect to total cell population (H/G: 0.22±0.21%; GCP: 0.65±0.52%, p=0.0004) and enriched lymphocyte population (H/G: 0.2±0.2%; GCP: 0.75±0.56%, p=0.0003) (Figure 2).

Results of mean fluorescence intensity (MFI) for CD161 from the CD4^+^CD161^+^ phenotype of the total cells were 24.1 and 22.8 for the H/G and GCP groups. The results for the enriched lymphocyte population were 23.8 and 22.7 for the H/G and GCP groups. The MFI for IL-23R from the Th17IL-23R^+^ phenotype of the total cells was 8.9 for the H/G group and 9.5 for the GCP group. In the enriched population, the values were 8.5 and 8.7. These results showed no statistically significant differences.

## Discussion

The Th17 subpopulation's key role in the pathogenesis of systemic inflammatory diseases has been extensively demonstrated. In rheumatoid arthritis, the number of Th17 cells increases with disease progression. Its presence is crucial in regulating cartilage and bone destruction due in part to the production of RANKL.^23^^,^^24^ In pathogenesis of multiple sclerosis, the proportion of Th1/Th17 cells determines the extent of inflammation in the brain and spinal cord.^25^ Th17 cells have been associated with the appearance and development of autoimmunity, infectious diseases and cancer.^26^ Nevertheless, not all Th17 cells are pathologic. Some populations of Th17 cells comprise regulatory subtypes and Th1-like cells and can be related to controlling disease dissemination and inflammation in systemic diseases and local infections of the periodontium. Determining the role of non-pathologic Th17 subpopulations is critical for establishing the prognosis of systemic inflammatory diseases in the presence of periodontitis.

Evidence shows that the proportion of Th17 cells may reflect the degree of systemic inflammation. This could make Th17 cells potential biomarkers of disease activity in patients with arthritis and the number of cells could help distinguish active disease stages^27^. However, in our study, the percentage of Th17 cells was not different between systemically healthy patients with and without active GCP. Nonetheless, GCP patients had a positive increase in cell populations for the IL-23 receptor in this study. This receptor is expressed in memory CD4^+^ and CD8^+^ cells as well as NKT cells.^4^ With respect to the Th17IL-23R^+^ subpopulation, not all Th17 cells respond to IL-23, but the proinflammatory role of Th17CD4^+^ cells that do respond to IL-23 is well established in many inflammatory disorders.^28^ IL-23 plays a key role as a mediator of endo-organic inflammation affecting the intestines, joints and central nervous system. Signaling via IL-23R promotes the generation of pathological Th17 as well as its contribution to several cellular functions including cytokine expansion, stabilization and secretion.^29^ Despite this, the role of IL-23 in Th17 functioning remains unclear and its role in differentiating this population is controversial. However, evidence suggests that IL-23 conditions the appearance of a proinflammatory phenotype.^28^

The results presented here indicate that, of the total Th17 cells, few can differentiate into a pathological profile (Th17IL-23R^+^). Low values of cells with this phenotype have been reported in the literature and vary between 0.42% and 2.69%.^6^^–^^9^ Proportions greater than these are considered as a result of chronic systemic inflammation, a product of an autoimmune process or tumor development.^11^^,^^12^ However, the significant increase in this pathological subpopulation in the presence of periodontitis may corroborate the impact of local infection on the systemic immune response mediated by this studied subpopulation. This study, to the best of our knowledge, is the first effort to further characterize the Th17 systemic response in periodontal infections (in otherwise systemically healthy individuals). These results should be further investigated by studying the different Th17 subprofiles by surface phenotyping and cytokine production.

It is also important to demonstrate the inter-individual differences in the expression levels of IL-23R and CD161 as these affect the response to IL-23 and therefore contribute to individual susceptibility during inflammatory processes. In chronic inflammatory diseases, and more specifically in autoimmunity, the CD161 fraction of memory T-cells is enriched with high IL-23R expression and is required for its inflammatory function.^30^ However, as not all Th17 cells are proinflammatory, an approximation of the Th17 pro or non-inflammatory phenotype was performed by analyzing the IL-23 and CD161 expression levels measured by the MFI. No statistically significant differences were found between the markers or the study groups. Although the MFI of IL-23R did not differ, these results suggest an increased proportion of Th17 cells with “pathological potential” in the peripheral blood of systemically healthy patients with chronic periodontitis, as well as in other inflammatory pathologies. Considering that no ongoing systemic inflammatory processes or autoimmune pathology were found in the patients included in this study by both clinical history and survey (Health Assessment Questionnaire, HAQ^21^^,^^22^) and by an absence of the rheumatoid factor,^6^^,^^8^ the increased Th17 cell counts are hypothesized to have originated in the inflamed periodontal tissue. This increase in the IL-23R^+^ population is extremely relevant because this receptor expression is related to stabilizing and maintaining the Th17 profile.^31^

One of the study limitations is the age difference between the periodontally healthy and periodontitis groups. Age can be a determining factor in the systemic immune response mediated by Th17 cells. Lee, et al.^32^ (2011) found a decreased frequency of Th17 memory cells and an increase in the differentiation of effector TH17 cells from naïve TCD4 cells in healthy adults over 65 years of age compared with young adults under 40 years of age. However, in this study, we found a differential increase in circulating Th17 cells that express the IL-23 receptor in systemically healthy individuals with significantly greater periodontal disease compared with the H/G group. There are no reports showing any relationship in the number of CD4IL-23R^+^ cells with age. Nonetheless, Shen, et al.^33^ (2013) report that the CD8CD161^+^ cells that express the IL-23 receptor decrease with age. Therefore, the increase in Th17IL-23R^+^ cells present in peripheral blood in subjects with periodontitis in this study may be associated with local infection rather than age.

Given the inflammatory nature of periodontitis and the evidence demonstrating that chronic periodontal infections can activate low-magnitude systemic inflammation, the mechanisms by which the immune system potentiates inflammatory processes and their regulatory mechanisms must be explored.

## Conclusion

Local infections may contribute to the pathogenesis of other diseases, the increased percentage of Th17IL-23R^+^ cells in systemically healthy patients with generalized chronic periodontitis found in this study could potentially constitute a biological marker for the progression of different systemic diseases in the presence of periodontitis. Also, such phenotype may also help to establish variations of this cellular subtype after periodontal therapy to control local infection in systemically healthy patients.
